# Ocular Biomechanics and Glaucoma

**DOI:** 10.3390/vision7020036

**Published:** 2023-04-23

**Authors:** Rodrigo Brazuna, Ruiz S. Alonso, Marcella Q. Salomão, Bruno F. Fernandes, Renato Ambrósio

**Affiliations:** 1 Department of Ophthalmology, Federal University of the State of Rio de Janeiro, Rio de Janeiro 22290-240, RJ, Brazil; 2 Department of Ophthalmology, Antonio Pedro University Hospital, Fluminense Federal University, Niterói 24033-900, RJ, Brazil; 3 Department of Ophthalmology, Federal University of São Paulo, São Paulo 04023-062, SP, Brazil; 4 Argumento Institute, Boucherville, QC J4B-2G6, Canada

**Keywords:** glaucoma, hysteresis, biomechanics, ORA, Corvis

## Abstract

Biomechanics is a branch of biophysics that deals with mechanics applied to biology. Corneal biomechanics have an important role in managing patients with glaucoma. While evidence suggests that patients with thin and stiffer corneas have a higher risk of developing glaucoma, it also influences the accurate measurement of intraocular pressure. We reviewed the pertinent literature to help increase our understanding of the biomechanics of the cornea and other ocular structures and how they can help optimize clinical and surgical treatments, taking into consideration individual variabilities, improve the diagnosis of suspected patients, and help monitor the response to treatment.

## 1. Introduction

Biomechanics is a branch of biophysics that deals with mechanics applied to biology in human or animal bodies. While biomechanics is especially concerned with the muscles and the skeleton, it is also used to refer to the functioning of any other part of the body, such as the cornea and other ocular structures [1]. The analysis of the corneal biomechanics has helped clinicians detect early or mild corneal ectasias [2,3,4], which can be further enhanced by integrating tomographic data acquired using the Pentacam (Oculus GmbH; Wetzlar, Germany) [3].

Corneal biomechanics also play a significant role in managing patients with glaucoma [5]. First, evidence suggests that eyes with thin and stiffer corneas are at a higher risk of developing glaucoma [6,7,8]. Second, corneal biomechanics affects and is affected by intraocular pressure (IOP) [9,10,11]. Therefore, one of the significant challenges of contemporary ophthalmology is understanding the independent contributions of corneal biomechanics and IOP to how the eye responds to mechanical stimuli and ensuring accurate measurements and proper monitoring of glaucoma patients [1].

## 2. The Ocular Response Analyzer (ORA)

**Figure 1:**
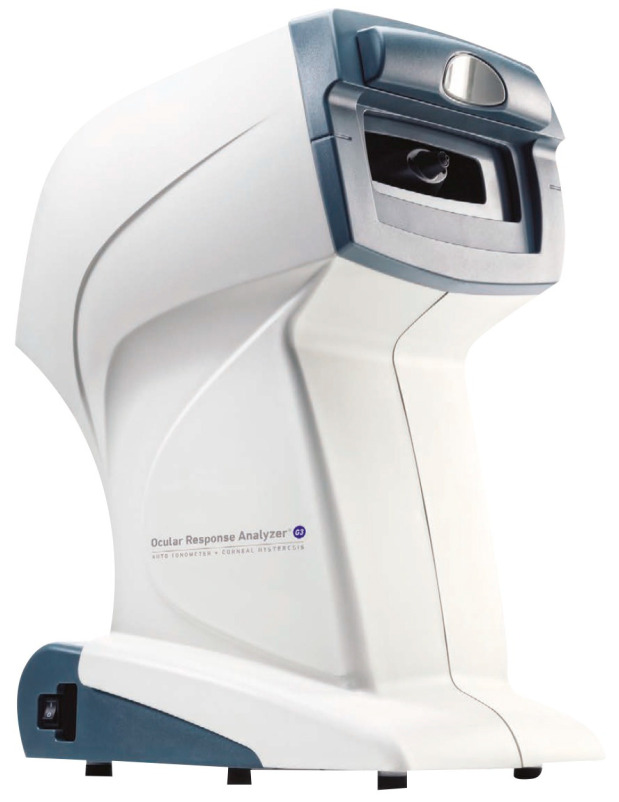
Ocular Response Analyzer tonometer (Image from Reichert Ophthalmic Instruments, Inc., Buffalo, NY, USA).

**Figure 2:**
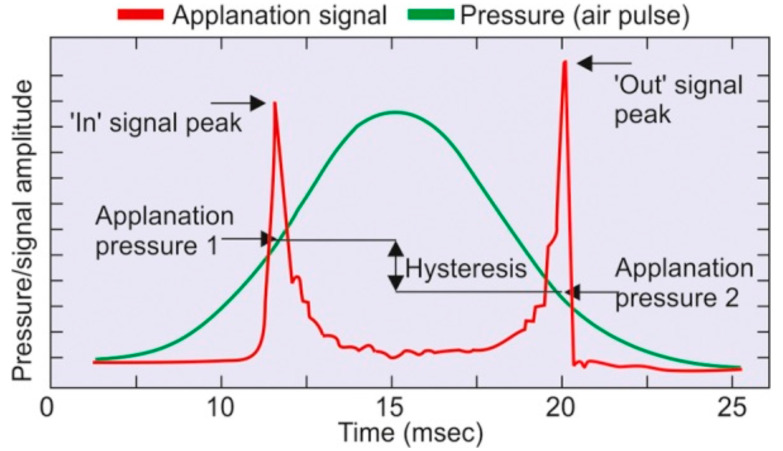
The deformation of the cornea resulting from the pressure of the air pulse as measured by the ORA. The parameters generated are corneal hysteresis (CH) and corneal resistance factor (CRF). Image from Kaushik S. et al. [21] (Creative Commons Attribution license).

The ORA (Reichert Ophthalmic Instruments, Inc., Buffalo, NY, USA) was introduced in 2005 by David Luce as the first instrument that allowed the in vivo assessment of ocular biomechanical properties (Figure 1) [12]. As a modified non-contact tonometer (NCT), it compensates for corneal biomechanics and is expected to provide a more accurate measurement of the IOP than the Goldmann applanation tonometer (GAT). The exam involves a fast jet of air that deforms the corneal curvature while the device records each moment of deformation. Initially, the cornea moves inwardly due to the pressure of the air jet until it reaches the first stage of applanation, at which point the first measurement of the IOP is taken (P1). The deformation of the cornea continues until a brief state of concavity occurs. Then the air pulse ends, and the cornea initiates the return to its normal convex shape. During this movement, the cornea passes through the second stage of applanation, when the second IOP measurement is taken (P2). The corneal hysteresis (CH) is calculated by the difference between P1 and P2 (Figure 2) and is also measured in mmHg [13]. The CH corresponds to a dissipation of energy during the loading and unloading phases, representing the viscoelastic characteristics of the entire globe, not exclusively of the cornea. In normal eyes, the mean ± SD CH is 10.7 ± 2.0 mm Hg, with a range of 6.1 to 17.6 mm Hg (SHAH). Studies have shown that CH is a dynamic parameter affected directly by the IOP with an inverse correlation. An increase in IOP decreases CH, and vice versa. A loading force on the cornea generates a response from the whole eye globe. As a consequence, it dissipates energy [14]. Studies have shown that CH will decrease with the stiffening of the sclera, confirming that CH is not a local corneal parameter and has no direct correspondence to corneal stiffness [15].

Other parameters generated by the ORA software are the corneal resistance factor (CRF), the compensated intraocular pressure (IOPcc), and the Goldmann-correlated IOP (IOPg). The CRF is a theoretical measure of the corneal elastic properties, calculated with the following formula to maximize the correlation with the central corneal thickness (CCT): *a* [P1 − 0.7P2] + *d*; *a* and *d* being calibration and regression constants [16]. The advantage of the IOPcc is that, compared to the IOP measured by the GAT, there is less influence from corneal structural properties such as the CCT [17]. Zhang et al. compared the measurement of the IOP by the ORA and GAT in post-refractive surgery eyes in a systematic review of the literature and found that the IOPcc is a closer measurement of the true IOP in eyes that underwent corneal procedures [18]. Lastly, the IOPg is the mean of the applanation pressures and is given by the formula IOPg = (P1 − P2)/2.

IOP is a constant force (loading) per unit area under the globe, playing an essential role in the biomechanical response [9,10,11]. The IOP is the most important predictor of deformation amplitude (DA) due to the pressure load of an air jet, followed by stiffness and thickness [19]. A stiffer response is expected in eyes with a greater IOP and a weaker cornea than in eyes with a lower IOP but a stronger cornea. The cornea and sclera have a non-linear stiffening response to an increase in IOP. Stress is defined as a force per unit of the cross-sectional area of a loaded, stretched tissue. On the other hand, strain is described as the non-dimensional deformation, or percent stretch, associated with the pulling of a certain tissue. The tangent elastic moduli are defined by the stress-strain slope curve at each value of strain and are also related to stiffness. The slope has a non-linear behavior: as the load increases, the slope increases too [20]. The stress distribution in the cornea can be calculated using the Hoop stress formula, σ = P · R/2t; σ being the stress, P the IOP, R the radius of curvature, and t the corneal thickness. We can conclude from this equation that thinner and flatter corneas are associated with more significant stress [20].

The biochemical properties of the sclera also contribute to the observed deformation of the cornea as a response to the air puff [19,22]. The biomechanical resistance of the sclera occurs after the aqueous displacement during the corneal recovery on the second applanation in air jet commercial tonometry. Studies have shown that the resistance to the movement of the aqueous humor is greater in eyes with stiffer scleras, which could be wrongly interpreted as a stiffer corneal deformation [23].

## 3. The Corvis ST Dynamic Scheimpflug Analyzer

**Figure 3:**
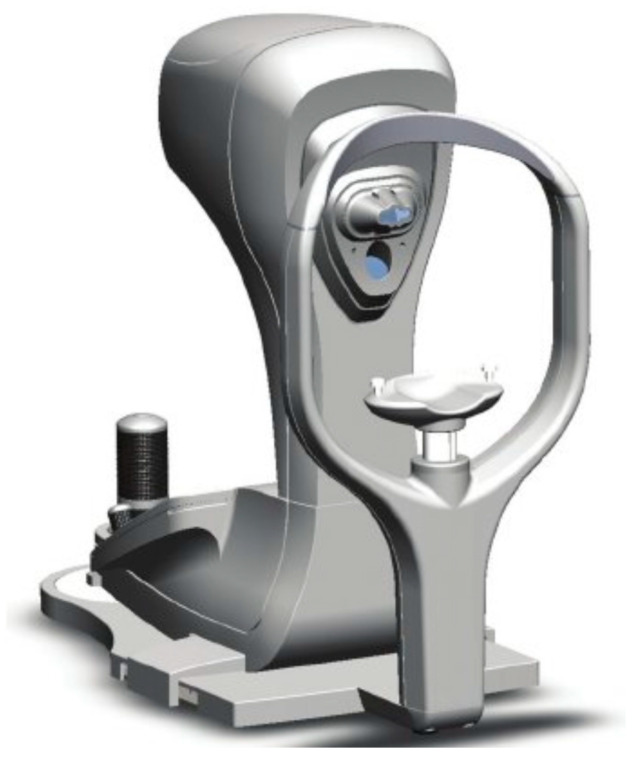
Oculus Corvis ST (Wetzlar, Germany).

Similar to the ORA, the Corvis ST (Oculus, Wetzlar, Germany) is a non-contact tonometer device in which an air jet deforms the cornea (Figure 3). However, the Corvis ST uses a collimated air pulse with a consistent pressure. The device uses an ultra-high-speed (UHS) Scheimpflug camera to acquire 4300 frames/s, covering 8.5 mm horizontally of a single slit, allowing a dynamic assessment of the induced deformation of the cornea [24].

**Figure 4:**
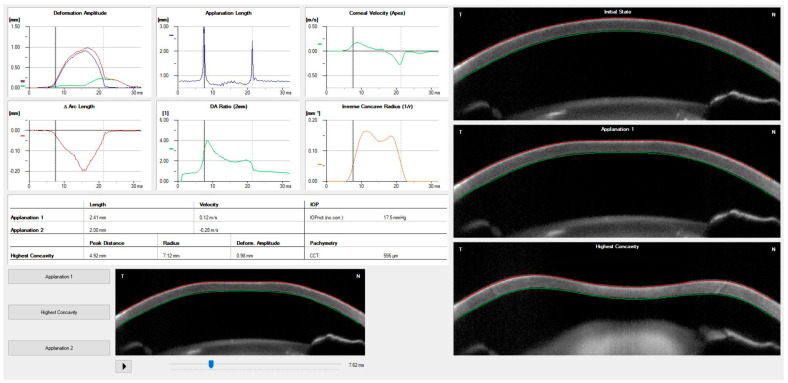
Corneal biomechanical properties and response measured by dynamic ultra-high-speed Scheimpflug imaging. The highest point concavity (HC) is shown in the image in the lower right corner. Personal archive.

With the air pulse, the cornea bends inward to the point of first applanation and continues until the point of highest concavity (HC) (Figure 4). Then the cornea recovers in the outward movement, and the cornea undergoes a second applanation point until finally returning to its natural state. The IOP is measured at the moment of the first corneal applanation moment, and the anterior and posterior limits of the cornea are identified by advanced algorithms. The Corvis ST also provides an array of metrics of corneal deformation based on the dynamic inspection of the corneal movement following the external pressure of the air jet, including the parameters that are extracted at the highest concavity point, such as the HC delta arc length and the HC deflection length [16,24].

Whole-eye movement (WEM) is a measure of the resistance of other ocular tissues taken at the moment the cornea reaches its maximal deformation and the pressure of the air jet increases. The orbital soft tissue and structure limit the WEM. The dynamic corneal response (DCR) parameters associated with the loading phase are naturally elastic [25]. One of these parameters is the point of highest concavity, which represents the moment of most significant resistance to aqueous movement. The stiffness parameter at the highest concavity (SP-HC) is the moment of capture of the scleral response calculated by the load at first applanation (air pressure, IOP) divided by the displacement from first applanation (A1) to the point of highest concavity [26]. The deformation ratio of 2 mm (DA ratio) and integrated inverse radius (IIR) are DCR parameters related to the deformation of the corneal shape during the exam, independent of IOP but associated with CCT. Other elastic DCR parameters related to the stiffness of the cornea are the stress-strain index (SSI) and the stiffness at the first applanation point (SP-A1) [26]. All the elastic parameters, such as the SSI, SP-A1, and SP-HC, are calculated with different algorithms, and their interpretation must be considered as different forms of stiffness. Decreasing the DA ratio, peak distance, and IIR is related to a higher resistance to change in the corneal shape and greater stiffness. A list of all deformation parameters that can be measured using the Corvis ST is presented in detail in Table 1.

**Figure 5:**
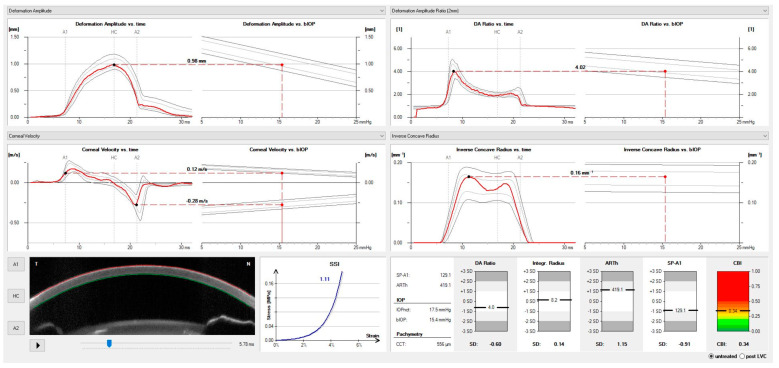
The Vinciguerra screening report shows the adjusted biomechanical intraocular pressure (bIOP), the Ambrósio Relational Thickness over the horizontal meridian (ARTh), and the Corvis Biomechanical Index (CBI). Personal archive.

The Corvis ST measures the IOP during the first applanation after the pressure from the air jet is applied [24]. The biomechanically compensated IOP (bIOP), available in the Vinciguerra Screening Report (Figure 5), includes the deformation response to provide another parameter for the IOP corrected through a finite element method using deformation data beyond CCT and age [28]. For the development of the bIOP algorithm, the analysis considered eyes with different variations of IOP (10–30 mmHg), CCT (445–645 microns), and age (30–90 years old). The corneal deformation response was predicted in each case and taken into consideration to estimate the Corvis IOP. The final analysis allowed the development of an algorithm that calculates the real IOP adjusted for Corvis IOP, CCT, and age. Subsequently, this algorithm of predictions of the corrected IOP was tested on a clinical data set consisting of a large number of normal eyes to investigate the association with corneal stiffness parameters, age, and CCT. Results demonstrated that the uncorrected IOP has a strong correlation with CCT but a weak correlation with age. However, the application of the algorithm to the IOP measurements yielded an IOP measurement that was less correlated with either CCT or age. The Vinciguerra screen enabled the calculation of indexes, including the Ambrósio Relational Thickness over the horizontal meridian (ARTh) and the Corvis Biomechanical Index (CBI), which help to discriminate between keratoconic and normal cases [29].

**Figure 6:**
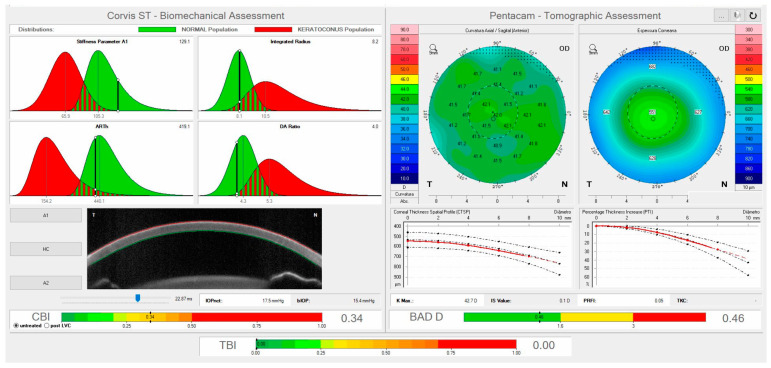
The ARV (Ambrosio, Roberts, and Vinciguerra) biomechanical and tomographic assessment shows the Tomographic Biomechanical Index (TBI) and the Corvis Biomechanical Index (CBI). Personal archive.

More recently, Ambrósio et al. applied artificial intelligence to combine tomographic and biomechanical data to develop the Tomographic Biomechanical Index (TBI). The TBI index demonstrated high sensitivity to diagnose mild or subclinical ectasia in cases of very asymmetric ectasia with normal tomographic maps (VAE-NT) (Figure 6) [30,31].

Ahmed et al. introduced a new intelligent algorithm of material stiffness for the in vivo assessment of the biomechanical characteristics of the human cornea: the Stress-Strain Index (SSI). The SSI showed a significant correlation with age but not a significant correlation with CCT or IOP [32]. Another study showed a possible association between the measurements obtained with the Corvis ST (CST) and CH. Patients with primary OAG and eyes from normal subjects were included in the study, and the patients were assessed in terms of CST metrics, ORA, axial length, average corneal curvature, CCT, and IOP with GAT. Parameters including DA (corneal softness), SP A1 (corneal stiffness), and Inverse Radius (integrated area under the curve of the inverse concave radius) were significantly correlated with CH. CST parameters were also significant but only weakly or moderately related to CH measures by ORA [33].

## 4. Hysteresis and Glaucoma

### 4.1. The Influence of Increased Stiffness of Ocular Structures in the Pathobiology of Glaucoma

Increased IOP and consequent glaucomatous damage have been associated with genetic factors, vascular abnormalities, metabolic changes, and immunologic factors [34,35,36,37]. However, the biomechanical characteristics of the ocular structures, which include the trabecular meshwork (TM), might also play a role. The juxtacanalicular connective tissue (JCT) is responsible for most of the resistance to the outflow of the aqueous humor (AH), and the inner wall endothelium pores account for only 10% at most. The spatial proximity of these two components causes a “funneling effect,” as the AH preferentially flows through the region of the JCT that is nearest to the inner wall pores, increasing up to 30-fold the JCT’s apparent resistance to flow. Despite the low flow resistance of the inner wall pores, alterations in the porosity of the inner wall can significantly affect the resistance to aqueous outflow [38,39]. Evidence shows that glaucomatous human trabecular meshwork (HTM) is stiffer than normal HTM, and modeling exercises suggest that it results in substantial impairment in the outflow. Biomechanical changes in the TM associated with ocular hypertension include increased rigidity, crosslinking, and extracellular matrix (ECM) deposition and may directly contribute to the onset of glaucoma and the progression of the disease [40,41].

The cellular-level deformation caused by biomechanical forces is determined by the stiffness, compressibility, and viscoelasticity of cells [42]. Compared to normal eyes, the TM of glaucomatous eyes has a 20-fold increase in stiffness, shows reduced cellularity, increased synthesis or deposition of ECM, higher actin contractility, the development of networks of cross-linked actin, and the dysregulation of multiple signaling pathways, including Wnt/β-catenin, TGFβ, and bone morphogenetic protein (BMP) pathways [43]. PIEZO is a stretch-sensitive ion channel present in the TM and retinal ganglion cells that aids in mechanotransduction by decreasing the contractibility of trabecular cells, facilitating AH outflow when activated [44,45]. TRV4 is another ion mechanosensitive channel that is involved in the remodeling of the TM by modulating RHO signaling [46]. Nitric oxide is a signal present in the endothelial cells of the Schlemm’s canal that decreases the resistance of the TM and the Schlemm’s canal, facilitating the AH passage [47]. Lastly, cytokines such as IL-8 and MCP-1 can cause an increase in the formation of actin stress fibers and focal adhesions, phosphorylation of the myosin light chain, and induce the contraction of TM cells, which combined can result in increased resistance to AH outflow [48].

The biomechanics of other ocular structures also have an important contribution to the pathogenesis of glaucoma. The sclera is an anatomic link between the cornea anteriorly and the lamina cribrosa posteriorly, and has a central role in the eye’s biomechanical effects [49]. The collagen in the scleral channel is distributed in a basket-weave shape, protecting the optic disc head’s axon and transferring tension to the equatorial sclera [50,51]. The differences in fibroblast morphology determine the sclera’s biomechanical response capacity [52]. Sulfated glycosaminoglycans (sGAGs) are related to increased scleral thickness and stiffness [53]. Although scleral stiffness is protective against glaucoma, ocular rigidity is not. Eyes with less elasticity are more susceptible to the deleterious effects of IOP variations [11,54]. The role of the sclera in glaucomatous eyes and its relationship with the lamina cribrosa are still unclear, as several studies continue to investigate whether scleral stiffness protects against IOP peaks, maintains the optic disc head and LC’s integrity, or contributes to glaucoma’s development [9,11,55,56].

Changes to the extracellular matrix (ECM) of the optic nerve head (ONH) and its relationship with resident axons and glial cells are associated with glaucoma, affecting the entire biomechanical response of the ONH. Therefore, the ONH is the primary site of glaucoma-related damage to axons [51,57]. Studies have shown that the radial connective fibers of the LC prevent the posterior displacement of the LC, while the circumferential and radial fibers decrease the strain inside the peripapillary scleral and neurological pre-laminar tissue [50]. These biomechanical anatomic characteristics help explain why the resistance of the inner layers of the sheath of the optic nerve is superior to that of the optic disc’s structures. Studies of the LC using optical coherence tomography or histology describe areas of missing connective tissue in the form of holes, gaps, pits, disinsertions, or irregularities. Since the connective tissue of the LC is believed to provide the structural support of adjacent neural tissues, its absence may increase the risk of neural tissue damage secondary to IOP fluctuations and consequent vision loss. Intriguingly, numerical models to study the effects of LC defects on neural tissue deformations caused by IOP variations showed that the partial loss of a beam can mitigate neural tissue insult related to IOP. These findings further support the idea that the architecture and biomechanics of the LC might be more multifaceted than merely protecting against IOP-induced damage to neural tissues [58]. The biological structures of the LC, episclera, and sclera are heterogeneous, which could, in part, explain the different patterns of damage in glaucoma or why different patients evolve to glaucoma at different IOP pressures [59].

The mechanisms involving fibroblastic changes, ECM remodeling, and their interplay with the biomechanical response in glaucoma are still not fully comprehended, and the field of knowledge in this area is continually growing [58].

### 4.2. Clinical Evidence on the Relationship between Hysteresis and Glaucoma

Although it is still debatable whether a stiffer globe contributes to glaucoma or rather is a consequence of the disease [20], there is growing evidence that supports an association between stiffer corneas and OAG and that patients with thin and stiffer corneas are at a higher risk of developing glaucoma [6,7,8,60]. Congdon et al. investigated 230 subjects in an observational study regarding the association of CH and CCT with visual perimeter damage and glaucoma progression risk and found CH, but not CCT, to be a predictive factor for the progression of visual field defects [61]. Another study suggested that CH and CRF, associated with CCT, could be risk factors for glaucoma [62]. Suzanna et al. showed that reduced hysteresis was linked to the risk of developing OAG, even when controlling for other risk factors such as IOP, CCT, field status, age, and the use of medical therapy for glaucoma. For each 1 mmHg reduction in CH, there was an associated 21% risk of patients with ocular hypertension converting to glaucoma [63]. The CH is a parameter that can also be affected by extrinsic factors, such as an increase observed after surgeries, some medical treatments, and laser procedures [64,65,66].

Biomechanical properties may not only provide valuable information to predict the progression of glaucoma but also have an influence on the response to IOP-lowering medications. The Ocular Hypertension Treatment Study showed that patients with a higher CCT presented more modest IOP reductions from IOP-reducing topical therapy [67]. Although these findings could not be replicated in the study by Agarwal et al., they investigated whether CH could also be a factor predictive of response to medical therapy, specifically prostaglandin analogs. There was a linear relationship between baseline CH and the absolute (r = 0.34, *p* = 0.01) and percent change (r = 0.31, *p* = 0.02) in IOP following therapy. Moreover, higher CH quintiles showed significantly smaller reductions in IOP compared with lower CH quartiles. Since it is known that patients with lower CH are at a higher risk of glaucoma progression, it is possible that although they are more responsive to topical PGA, they might also need greater IOP reductions to avoid the progression of glaucomatous damage [64].

Unlike CCT, the CH is a dynamic parameter that changes with age, surgeries, prostaglandin treatment, and IOP [20]. While CCT and CH are positively related, there is a negative relationship between IOP and CH [68]. Specifically, thick corneas are able to dissipate energy better than thinner ones, and eyes with higher IOP tend to be less efficient at dissipating energy. The age-related thinning of the cornea happens slowly over time, which explains why it causes a lower impact on the CH than the acute variations of IOP [62,69,70]. It is also important to note that an increase in CCT caused by corneal edema is associated with a lower CH, stressing the importance of considering all parameters and not only CCT [5].

Two randomized clinical trials, the Collaborative Initial Glaucoma Treatment Study and the Advanced Glaucoma Intervention Study, have suggested that the progression of visual field defects in OAG is connected with high IOP variations [71,72]. These studies indicate that transiently high variations in IOP may expand the scleral channel, increasing strain in the lamina cribrosa and causing axon damage. The biomechanical capacity of the biomechanics properties of the entire eye, such as the optic disc and the connective tissue within the scleral channel, and the consequent ability to dissipate energy and preserve RGC axons could explain the individual variability in responding to IOP variations [55,73]. For example, age and CH are two parameters that are related to the displacement of the LC, which is known to absorb IOP fluctuations and protect eyes with glaucoma from disease progression [9]. Considering that CH is the ability of not only the cornea but the whole globe to dampen energy, a lower CH has been associated with visual field loss in OAG and normal tension glaucoma [74,75]. The CH is indeed the most important predictor compared to other metrics such as CRF, CCT, corneal-compensated IOP, Goldmann-correlated IOP, and refractive error [74].

## 5. DCR Parameters and Glaucoma

Interestingly, other studies did not show any differences in corneal mechanics between glaucoma patients and healthy controls, and some even suggested that glaucoma patients, in fact, have more deformable corneas [76,77,78]. A plausible explanation is using incorrect Corvis ST parameters to evaluate corneal stiffness. Another reason for these differences may be the selection bias of patients in prostaglandin treatment, which leads to the consequent lowering of the stiffness of the entire globe. The binding of analogs of prostaglandins to F-receptors on the cornea, TM, ciliary body, episclera, and sclera likely explains the increase of the CH [79,80,81]. This mechanism activates the F-receptors, increasing extracellular matrix expansion and reducing collagen [82]. The remodeling of these structures decreases the resistance of aqueous flow at the uveoscleral outflow pathway and possibly increases the capacity of the entire globe to dissipate energy [83,84]. The fact that the change in corneal compensated IOP from PGA therapy persists for 6 weeks after the cessation of therapy casts doubt on whether the structural alterations induced by PGA therapy are reversible [85].

One new parameter that evaluates scleral stiffness is the SP-HC, although its relationship with the risk of glaucoma is still unclear. Vinciguerra et al. showed that a low SP-HC is related to advanced visual field loss in OAG. However, one of the limitations of the study is that a significant number of glaucoma patients included in the analysis were treated with analogs of prostaglandins, which are known to decrease corneal stiffness [73,86]. Other researchers also studied patients under therapy for glaucoma but could not show a link between SP-HC and a higher risk of glaucoma [7]. For that reason, further studies are warranted to clarify the importance of SP-HC in OAG, glaucoma suspects, and healthy patients and its relationship with CH and other conditions that change ocular rigidity, like aging and race. Studies using the ORA have suggested that waveform parameters related to the shape of the second peak are associated with scleral stiffness. This new approach may help us evaluate and understand in vivo the association between scleral stiffness and glaucoma [87].

## 6. Conclusions

Understanding the biomechanics of the eye can help clinicians detect early or mild corneal ectasias, but it can also provide valuable insights regarding the pathogenesis of glaucomatous damage, improve the diagnosis of suspected patients, and help monitor the response to IOP-lowering therapies and surgical interventions. The multitude of parameters available allows a thorough analysis and identification of the unique biomechanical characteristics of each patient and their eyes. It will also enable a clearer understanding of the effects of procedures that mechanically interact or interfere with the eye, including keratoconus risk profiling, refractive surgery planning, and optimization of different collagen crosslinking treatment protocols. Moreover, a more accurate measurement of the IOP allows for a better approach to diagnosing and managing patients with glaucoma and the optimization of clinical and surgical treatments [88,89].

## Figures and Tables

**Table 1:** Corneal deformation parameters provided by the Corvis ST (adapted from Brazuna R. et al. [27], Creative Commons Attribution (CC BY) license).

Corvis ST Parameter	Definition
1st Applanation	The first applanation of the cornea during the air puff (in ms). The length of the applanation at this moment appears in parenthesis (in mm).
Highest Concavity	The instant that the cornea assumes its maximum concavity during the air puff (in ms). The length of the distance between the two peaks of the cornea at this moment appears in parenthesis (in mm).
2nd Applanation	The second applanation of the cornea during the air puff (in milliseconds). The length of the applanation at this moment appears in parenthesis (in mm).
Maximum Deformation	The amount (in mm) of the maximum corneal deformation during the air puff.
Wing Distance	The length of the distance between the two peaks of the cornea at this instant (in mm).
Maximum Velocity (in)	The maximum velocity during the ingoing phase (in m/s).
Maximum Velocity	The maximum velocity during the outgoing phase (in m/s).
Curvature Radius Normal	The cornea in its natural state has a radius of curvature (in mm).
Curvature Radius HC	The corneal radius of curvature at the time of maximum concavity during the air puff (in mm).
Cornea Thickness	Measurement of the corneal thickness (in mm).
IOP	Measurement of the intraocular pressure (in mmHg).
bIOP	Biomechanically-corrected IOP.
DA ratio Max (Deformation amplitude ratio max. 2 mm)	Ratio between the deformation amplitude at the apex and the average deformation amplitude measured at 2 mm from the center.
ARth (Ambrósio’s relational thickness to the horizontal profile)	Describes the thickness profile in the temporal-nasal direction and is defined as corneal thickness from thinnest to pachymetric progression.
SP-A1(Stiffness parameter at A1)	Describes corneal stiffness as defined by resultant pressure (Pr) divided by deflection amplitude at A1.
SP-HC	Corneal stiffness at the highest concavity point.
TBI (Tomographic biomechanical index)	Index that combines tomographic and biomechanical data for keratoconus detection.
BGF (Biomechanical Glaucoma factor)	Independent risk indicator for normal tension glaucoma.
SSI (Stress-strain index)	Index that indicates the position of the stress-strain curves. Less dependent on corneal thickness and IOP.
CBI (Corvis biomechanical index)	Overall biomechanical index for keratoconus detection.
Whole eye movement (WEM)	The entire globe’s movement after the cornea passes its limits during the jet air pulse is resisted by the orbital structures.
Deformation Amplitude (DA)	The movement of the corneal deformation from the apex to the highest concavity.
Deflection amplitude (DeflA)	The difference between the DA and the WEM.
HC dArc length	Change in arc length during the highest concavity moment from the initial state, in a defined 7-mm zone.
HC deflection length	Length of the flattened cornea at its highest concavity.

## Data Availability

Not applicable.

## References

[1] Liu J., Roberts C.J. (2005). Influence of corneal biomechanical properties on intraocular pressure measurement: Quantitative analysis. J. Cataract. Refract. Surg..

[2] Fontes B.M., Ambrosio R., Jardim D., Velarde G.C., Nose W. (2010). Corneal biomechanical metrics and anterior segment parameters in mild keratoconus. Ophthalmology.

[3] Luz A., Fontes B.M., Lopes B., Ramos I., Schor P., Ambrosio R. (2013). ORA waveform-derived biomechanical parameters to distinguish normal from keratoconic eyes. Arq. Bras. Oftalmol..

[4] Ventura B.V., Machado A.P., Ambrosio R., Ribeiro G., Araujo L.N., Luz A., Lyra J.M. (2013). Analysis of waveform-derived ORA parameters in early forms of keratoconus and normal corneas. J. Refract. Surg..

[5] da Silva J.A.S., da Silva R.S., Ambrósio R. (2012). Relevância da biomecânica da córnea no glaucoma. Rev. Bras. Oftalmol..

[6] Catania F., Morenghi E., Rosetta P., Paolo V., Vinciguerra R. (2023). Corneal Biomechanics Assessment with Ultra High Speed Scheimpflug Camera in Primary Open Angle Glaucoma Compared with Healthy Subjects: A meta-analysis of the Literature. Curr. Eye Res..

[7] Qassim A., Mullany S., Abedi F., Marshall H., Hassall M.M., Kolovos A., Knight L.S.W., Nguyen T., Awadalla M.S., Chappell A. (2021). Corneal Stiffness Parameters Are Predictive of Structural and Functional Progression in Glaucoma Suspect Eyes. Ophthalmology.

[8] Salvetat M.L., Zeppieri M., Tosoni C., Felletti M., Grasso L., Brusini P. (2015). Corneal Deformation Parameters Provided by the Corvis-ST Pachy-Tonometer in Healthy Subjects and Glaucoma Patients. J. Glaucoma.

[9] Lanzagorta-Aresti A., Perez-Lopez M., Palacios-Pozo E., Davo-Cabrera J. (2017). Relationship between corneal hysteresis and lamina cribrosa displacement after medical reduction of intraocular pressure. Br. J. Ophthalmol..

[10] Zeimer R.C., Ogura Y. (1989). The relation between glaucomatous damage and optic nerve head mechanical compliance. Arch. Ophthalmol..

[11] Kimball E.C., Nguyen C., Steinhart M.R., Nguyen T.D., Pease M.E., Oglesby E.N., Oveson B.C., Quigley H.A. (2014). Experimental scleral cross-linking increases glaucoma damage in a mouse model. Exp. Eye Res..

[12] Luce D.A. (2005). Determining in vivo biomechanical properties of the cornea with an ocular response analyzer. J. Cataract. Refract. Surg..

[13] Brandt J.D. (2007). Central corneal thickness, tonometry, and glaucoma risk—A guide for the perplexed. Can. J. Ophthalmol..

[14] Roberts C.J. (2021). Corneal hysteresis and beyond: Does it involve the sclera?. J. Cataract. Refract. Surg..

[15] Taroni L., Bernabei F., Pellegrini M., Roda M., Toschi P.G., Mahmoud A.M., Schiavi C., Giannaccare G., Roberts C.J. (2020). Corneal Biomechanical Response Alteration After Scleral Buckling Surgery for Rhegmatogenous Retinal Detachment. Am. J. Ophthalmol..

[16] Roberts C.J. (2014). Concepts and misconceptions in corneal biomechanics. J. Cataract. Refract. Surg..

[17] Hager A., Schroeder B., Sadeghi M., Grossherr M., Wiegand W. (2007). The influence of corneal hysteresis and corneal resistance factor on the measurement of intraocular pressure. Ophthalmologe.

[18] Zhang H., Sun Z., Li L., Sun R., Zhang H. (2020). Comparison of intraocular pressure measured by ocular response analyzer and Goldmann applanation tonometer after corneal refractive surgery: A systematic review and meta-analysis. BMC Ophthalmol..

[19] Nguyen B.A., Roberts C.J., Reilly M.A. (2018). Biomechanical Impact of the Sclera on Corneal Deformation Response to an Air-Puff: A Finite-Element Study. Front. Bioeng. Biotechnol..

[20] Yuhas P.T., Roberts C.J. (2023). Clinical Ocular Biomechanics: Where Are We after 20 Years of Progress?. Curr. Eye Res..

[21] Kaushik S., Pandav S.S. (2021). Ocular Response Analyzer. J. Curr. Glaucoma. Pract..

[22] Nguyen B.A., Reilly M.A., Roberts C.J. (2020). Biomechanical contribution of the sclera to dynamic corneal response in air-puff induced deformation in human donor eyes. Exp. Eye Res..

[23] Metzler K.M., Mahmoud A.M., Liu J., Roberts C.J. (2014). Deformation response of paired donor corneas to an air puff: Intact whole globe versus mounted corneoscleral rim. J. Cataract. Refract. Surg..

[24] Ambrósio R., Ramos I., Luz A., Faria F.C., Andreas S., Krug M., Belin M.W., Roberts C.J. (2013). Dynamic ultra-high speed Scheimpflug imaging for assessing corneal biomechanical properties. Rev. Bras. Oftalmol..

[25] Leszczynska A., Moehler K., Spoerl E., Ramm L., Herber R., Pillunat L.E., Terai N. (2018). Measurement of Orbital Biomechanical Properties in Patients with Thyroid Orbitopathy Using the Dynamic Scheimpflug Analyzer (Corvis ST). Curr. Eye Res..

[26] Roberts C.J., Mahmoud A.M., Bons J.P., Hossain A., Elsheikh A., Vinciguerra R., Vinciguerra P., Ambrosio R. (2017). Introduction of Two Novel Stiffness Parameters and Interpretation of Air Puff-Induced Biomechanical Deformation Parameters With a Dynamic Scheimpflug Analyzer. J. Refract. Surg..

[27] Brazuna R., Salomão M., Esporcatte B., Macedo M., Esporcatte L., Colombini G.N., Ambrósio R. (2022). Corneal Biomechanics and Glaucoma Beyond the Bidirectional Impact of Intraocular Pressure and Corneal Deformation Response. Rev. Bras. Oftalmol..

[28] Joda A.A., Shervin M.M., Kook D., Elsheikh A. (2016). Development and validation of a correction equation for Corvis tonometry. Comput. Methods Biomech. Biomed. Eng..

[29] Vinciguerra R., Ambrosio R., Elsheikh A., Roberts C.J., Lopes B., Morenghi E., Azzolini C., Vinciguerra P. (2016). Detection of Keratoconus With a New Biomechanical Index. J. Refract. Surg..

[30] Ambrosio R., Lopes B.T., Faria-Correia F., Salomao M.Q., Buhren J., Roberts C.J., Elsheikh A., Vinciguerra R., Vinciguerra P. (2017). Integration of Scheimpflug-Based Corneal Tomography and Biomechanical Assessments for Enhancing Ectasia Detection. J. Refract. Surg..

[31] Sedaghat M.R., Momeni-Moghaddam H., Ambrosio R., Roberts C.J., Yekta A.A., Danesh Z., Reisdorf S., Khabazkhoob M., Heidari H.R., Sadeghi J. (2018). Long-term Evaluation of Corneal Biomechanical Properties After Corneal Cross-linking for Keratoconus: A 4-Year Longitudinal Study. J. Refract. Surg..

[32] Eliasy A., Chen K.J., Vinciguerra R., Lopes B.T., Abass A., Vinciguerra P., Ambrosio R., Roberts C.J., Elsheikh A. (2019). Determination of Corneal Biomechanical Behavior in-vivo for Healthy Eyes Using CorVis ST Tonometry: Stress-Strain Index. Front. Bioeng. Biotechnol..

[33] Fujishiro T., Matsuura M., Fujino Y., Murata H., Tokumo K., Nakakura S., Kiuchi Y., Asaoka R. (2020). The Relationship Between Corvis ST Tonometry Parameters and Ocular Response Analyzer Corneal Hysteresis. J. Glaucoma.

[34] Wiggs J.L., Pasquale L.R. (2017). Genetics of glaucoma. Hum. Mol. Genet..

[35] Wareham L.K., Calkins D.J. (2020). The Neurovascular Unit in Glaucomatous Neurodegeneration. Front. Cell Dev. Biol..

[36] Williams P.A., Harder J.M., John S.W.M. (2017). Glaucoma as a Metabolic Optic Neuropathy: Making the Case for Nicotinamide Treatment in Glaucoma. J. Glaucoma.

[37] Bell K., Funke S., Grus F.H. (2019). Autoimmunity and glaucoma. Ophthalmologe.

[38] Grant W.M. (1951). Clinical measurements of aqueous outflow. Am. J. Ophthalmol..

[39] Johnson M., Shapiro A., Ethier C.R., Kamm R.D. (1992). Modulation of outflow resistance by the pores of the inner wall endothelium. Investig. Ophthalmol. Vis. Sci..

[40] Last J.A., Pan T., Ding Y., Reilly C.M., Keller K., Acott T.S., Fautsch M.P., Murphy C.J., Russell P. (2011). Elastic modulus determination of normal and glaucomatous human trabecular meshwork. Investig. Ophthalmol. Vis. Sci..

[41] Yemanyi F., Raghunathan V. (2020). Lysophosphatidic Acid and IL-6 Trans-signaling Interact via YAP/TAZ and STAT3 Signaling Pathways in Human Trabecular Meshwork Cells. Investig. Ophthalmol. Vis. Sci..

[42] Ethier C.R., Simmons C.A. (2007). Introductory Biomechanics: From Cells to Organisms.

[43] Dhamodaran K., Baidouri H., Sandoval L., Raghunathan V. (2020). Wnt Activation After Inhibition Restores Trabecular Meshwork Cells Toward a Normal Phenotype. Investig. Ophthalmol. Vis. Sci..

[44] Yarishkin O., Phuong T.T.T., Baumann J.M., De Ieso M.L., Vazquez-Chona F., Rudzitis C.N., Sundberg C., Lakk M., Stamer W.D., Križaj D. (2021). Piezo1 channels mediate trabecular meshwork mechanotransduction and promote aqueous fluid outflow. J. Physiol..

[45] Zhu W., Hou F., Fang J., Bahrani Fard M.R., Liu Y., Ren S., Wu S., Qi Y., Sui S., Read A.T. (2021). The role of Piezo1 in conventional aqueous humor outflow dynamics. iScience.

[46] Lakk M., Križaj D. (2021). TRPV4-Rho signaling drives cytoskeletal and focal adhesion remodeling in trabecular meshwork cells. Am. J. Physiol. Cell Physiol..

[47] Madekurozwa M., Stamer W.D., Reina-Torres E., Sherwood J.M., Overby D.R. (2021). The ocular pulse decreases aqueous humor outflow resistance by stimulating nitric oxide production. Am. J. Physiol. Cell Physiol..

[48] Lee J., Choi J.A., Ju H.H., Kim J.E., Paik S.Y., Rao P.V. (2021). Role of MCP-1 and IL-8 in viral anterior uveitis, and contractility and fibrogenic activity of trabecular meshwork cells. Sci. Rep..

[49] Sigal I.A., Flanagan J.G., Ethier C.R. (2005). Factors influencing optic nerve head biomechanics. Investig. Ophthalmol. Vis. Sci..

[50] Hua Y., Voorhees A.P., Jan N.J., Wang B., Waxman S., Schuman J.S., Sigal I.A. (2020). Role of radially aligned scleral collagen fibers in optic nerve head biomechanics. Exp. Eye Res..

[51] Safa B.N., Wong C.A., Ha J., Ethier C.R. (2022). Glaucoma and biomechanics. Curr. Opin. Ophthalmol..

[52] Szeto J., Chow A., McCrea L., Mozzer A., Nguyen T.D., Quigley H.A., Pitha I. (2021). Regional Differences and Physiologic Behaviors in Peripapillary Scleral Fibroblasts. Investig. Ophthalmol. Vis. Sci..

[53] Murienne B.J., Chen M.L., Quigley H.A., Nguyen T.D. (2016). The contribution of glycosaminoglycans to the mechanical behaviour of the posterior human sclera. J. R. Soc. Interface.

[54] Morris H.J., Tang J., Cruz Perez B., Pan X., Hart R.T., Weber P.A., Liu J. (2013). Correlation between biomechanical responses of posterior sclera and IOP elevations during micro intraocular volume change. Investig. Ophthalmol. Vis. Sci..

[55] Sigal I.A., Yang H., Roberts M.D., Grimm J.L., Burgoyne C.F., Demirel S., Downs J.C. (2011). IOP-induced lamina cribrosa deformation and scleral canal expansion: Independent or related?. Investig. Ophthalmol. Vis. Sci..

[56] Coudrillier B., Campbell I.C., Read A.T., Geraldes D.M., Vo N.T., Feola A., Mulvihill J., Albon J., Abel R.L., Ethier C.R. (2016). Effects of Peripapillary Scleral Stiffening on the Deformation of the Lamina Cribrosa. Investig. Ophthalmol. Vis. Sci..

[57] Quigley H.A., Addicks E.M. (1981). Regional differences in the structure of the lamina cribrosa and their relation to glaucomatous optic nerve damage. Arch. Ophthalmol..

[58] Voorhees A.P., Hua Y., Brazile B.L., Wang B., Waxman S., Schuman J.S., Sigal I.A. (2020). So-Called Lamina Cribrosa Defects May Mitigate IOP-Induced Neural Tissue Insult. Investig. Ophthalmol. Vis. Sci..

[59] Eilaghi A., Flanagan J.G., Simmons C.A., Ethier C.R. (2010). Effects of scleral stiffness properties on optic nerve head biomechanics. Ann. Biomed. Eng..

[60] Wong B.J., Moghimi S., Zangwill L.M., Christopher M., Belghith A., Ekici E., Bowd C., Fazio M.A., Girkin C.A., Weinreb R.N. (2020). Relationship of Corneal Hysteresis and Anterior Lamina Cribrosa Displacement in Glaucoma. Am. J. Ophthalmol..

[61] Congdon N.G., Broman A.T., Bandeen-Roche K., Grover D., Quigley H.A. (2006). Central corneal thickness and corneal hysteresis associated with glaucoma damage. Am. J. Ophthalmol..

[62] De Moraes C.V., Hill V., Tello C., Liebmann J.M., Ritch R. (2012). Lower corneal hysteresis is associated with more rapid glaucomatous visual field progression. J. Glaucoma.

[63] Susanna C.N., Diniz-Filho A., Daga F.B., Susanna B.N., Zhu F., Ogata N.G., Medeiros F.A. (2018). A Prospective Longitudinal Study to Investigate Corneal Hysteresis as a Risk Factor for Predicting Development of Glaucoma. Am. J. Ophthalmol..

[64] Agarwal D.R., Ehrlich J.R., Shimmyo M., Radcliffe N.M. (2012). The relationship between corneal hysteresis and the magnitude of intraocular pressure reduction with topical prostaglandin therapy. Br. J. Ophthalmol..

[65] Hirneiss C., Sekura K., Brandlhuber U., Kampik A., Kernt M. (2013). Corneal biomechanics predict the outcome of selective laser trabeculoplasty in medically uncontrolled glaucoma. Graefes. Arch. Clin. Exp. Ophthalmol..

[66] Sun L., Shen M., Wang J., Fang A., Xu A., Fang H., Lu F. (2009). Recovery of corneal hysteresis after reduction of intraocular pressure in chronic primary angle-closure glaucoma. Am. J. Ophthalmol..

[67] Brandt J.D., Beiser J.A., Gordon M.O., Kass M.A., Ocular Hypertension Treatment Study G. (2004). Central corneal thickness and measured IOP response to topical ocular hypotensive medication in the Ocular Hypertension Treatment Study. Am. J. Ophthalmol..

[68] Kotecha A., Elsheikh A., Roberts C.R., Zhu H., Garway-Heath D.F. (2006). Corneal thickness- and age-related biomechanical properties of the cornea measured with the ocular response analyzer. Investig. Ophthalmol. Vis. Sci..

[69] Brandt J.D., Gordon M.O., Beiser J.A., Lin S.C., Alexander M.Y., Kass M.A., Ocular Hypertension Treatment Study G. (2008). Changes in central corneal thickness over time: The ocular hypertension treatment study. Ophthalmology.

[70] Sullivan-Mee M., Katiyar S., Pensyl D., Halverson K.D., Qualls C. (2012). Relative importance of factors affecting corneal hysteresis measurement. Optom. Vis. Sci..

[71] Musch D.C., Gillespie B.W., Niziol L.M., Lichter P.R., Varma R., Group C.S. (2011). Intraocular pressure control and long-term visual field loss in the Collaborative Initial Glaucoma Treatment Study. Ophthalmology.

[72] Nouri-Mahdavi K., Hoffman D., Coleman A.L., Liu G., Li G., Gaasterland D., Caprioli J., Advanced Glaucoma Intervention S. (2004). Predictive factors for glaucomatous visual field progression in the Advanced Glaucoma Intervention Study. Ophthalmology.

[73] Vinciguerra R., Rehman S., Vallabh N.A., Batterbury M., Czanner G., Choudhary A., Cheeseman R., Elsheikh A., Willoughby C.E. (2020). Corneal biomechanics and biomechanically corrected intraocular pressure in primary open-angle glaucoma, ocular hypertension and controls. Br. J. Ophthalmol..

[74] Anand A., De Moraes C.G., Teng C.C., Tello C., Liebmann J.M., Ritch R. (2010). Corneal hysteresis and visual field asymmetry in open angle glaucoma. Investig. Ophthalmol. Vis. Sci..

[75] Helmy H., Leila M., Zaki A.A. (2016). Corneal biomechanics in asymmetrical normal-tension glaucoma. Clin. Ophthalmol..

[76] Miki A., Yasukura Y., Weinreb R.N., Maeda N., Yamada T., Koh S., Asai T., Ikuno Y., Nishida K. (2020). Dynamic Scheimpflug Ocular Biomechanical Parameters in Untreated Primary Open Angle Glaucoma Eyes. Investig. Ophthalmol. Vis. Sci..

[77] Pradhan Z.S., Deshmukh S., Dixit S., Sreenivasaiah S., Shroff S., Devi S., Webers C.A.B., Rao H.L. (2020). A comparison of the corneal biomechanics in pseudoexfoliation glaucoma, primary open-angle glaucoma and healthy controls using Corvis ST. PLoS ONE.

[78] Silva N., Ferreira A., Baptista P.M., Figueiredo A., Reis R., Sampaio I., Beirao J., Vinciguerra R., Meneres P., Meneres M.J. (2022). Corneal Biomechanics for Ocular Hypertension, Primary Open-Angle Glaucoma, and Amyloidotic Glaucoma: A Comparative Study by Corvis ST. Clin. Ophthalmol..

[79] Schlotzer-Schrehardt U., Zenkel M., Nusing R.M. (2002). Expression and localization of FP and EP prostanoid receptor subtypes in human ocular tissues. Investig. Ophthalmol. Vis. Sci..

[80] Sharif N.A., Kelly C.R., Crider J.Y., Williams G.W., Xu S.X. (2003). Ocular hypotensive FP prostaglandin (PG) analogs: PG receptor subtype binding affinities and selectivities, and agonist potencies at FP and other PG receptors in cultured cells. J. Ocul. Pharmacol. Ther..

[81] Zheng X., Wang Y., Zhao Y., Cao S., Zhu R., Huang W., Yu A., Huang J., Wang Q., Wang J. (2019). Experimental Evaluation of Travoprost-Induced Changes in Biomechanical Behavior of Ex-Vivo Rabbit Corneas. Curr. Eye Res..

[82] Weinreb R.N., Toris C.B., Gabelt B.T., Lindsey J.D., Kaufman P.L. (2002). Effects of prostaglandins on the aqueous humor outflow pathways. Surv. Ophthalmol..

[83] Kim J.W., Lindsey J.D., Wang N., Weinreb R.N. (2001). Increased human scleral permeability with prostaglandin exposure. Investig. Ophthalmol. Vis. Sci..

[84] Lindsey J.D., Crowston J.G., Tran A., Morris C., Weinreb R.N. (2007). Direct matrix metalloproteinase enhancement of transscleral permeability. Investig. Ophthalmol. Vis. Sci..

[85] Meda R., Wang Q., Paoloni D., Harasymowycz P., Brunette I. (2017). The impact of chronic use of prostaglandin analogues on the biomechanical properties of the cornea in patients with primary open-angle glaucoma. Br. J. Ophthalmol..

[86] Scott J.A., Roberts C.J., Mahmoud A.M., Jain S.G. (2021). Evaluating the Relationship of Intraocular Pressure and Anterior Chamber Volume with Use of Prostaglandin Analogues. J. Glaucoma.

[87] Aoki S., Murata H., Matsuura M., Fujino Y., Nakakura S., Nakao Y., Kiuchi Y., Asaoka R. (2018). The Relationship between the Waveform Parameters from the Ocular Response Analyzer and the Progression of Glaucoma. Ophthalmol. Glaucoma.

[88] Faria-Correia F., Ramos I., Valbon B., Luz A., Roberts C.J., Ambrosio R. (2013). Scheimpflug-based tomography and biomechanical assessment in pressure-induced stromal keratopathy. J. Refract. Surg..

[89] Goldich Y., Marcovich A.L., Barkana Y., Mandel Y., Hirsh A., Morad Y., Avni I., Zadok D. (2012). Clinical and corneal biomechanical changes after collagen cross-linking with riboflavin and UV irradiation in patients with progressive keratoconus: Results after 2 years of follow-up. Cornea.

